# Is zero underestimation feasible? Extended Vacuum-assisted breast biopsy in solid lesions – a blind study

**DOI:** 10.1186/1477-7819-5-53

**Published:** 2007-05-14

**Authors:** George C Zografos, Flora Zagouri, Theodoros N Sergentanis, Dimitra Koulocheri, Afroditi Nonni, Vassiliki Oikonomou, Philip Domeyer, Maria Kotsani, Constantine Fotiadis, John Bramis

**Affiliations:** 1 1st Department of surgery, School of Medicine, Athens University, Greece; 2 Department of Radiology, School of Medicine, Athens University, Greece; 3 Department of Pathology, School of Medicine, Athens University, Greece; 4 Department of Cytology, School of Medicine, Athens University, Greece

## Abstract

**Background:**

Vacuum-Assisted Breast Biopsy (VABB) is effective for the preoperative diagnosis of non-palpable mammographic solid lesions. The main disadvantage is underestimation, which might render the management of atypical ductal hyperplasia (ADH), and ductal carcinoma in situ (DCIS) difficult. This study aims to develop and assess a modified way of performing VABB.

**Patients and methods:**

A total of 107 women with non-palpable mammographic breast solid tumors BI-RADS 3 and 4 underwent VABB with 11G, on the stereotactic Fischer's table. 54 women were allocated to the recommended protocol and 24 cores were obtained according to the consensus meeting in Nordesterdt (1 offset-main target in the middle of the lesion and one offset inside). 53 women were randomly allocated to the extended protocol and 96 cores were excised (one offset-main target in the middle of the lesion and 7 peripheral offsets). A preoperative diagnosis was established. Women with a preoperative diagnosis of precursor/preinvasive/invasive lesion underwent open surgery. A second pathologist, blind to the preoperative results and to the protocol made the postoperative diagnosis. The percentage of the surface excised via VABB was retrospectively calculated on the mammogram. The discrepancy between preoperative and postoperative diagnoses along with the protocol adopted and the volume removed were evaluated by Fisher's exact test and Mann-Whitney-Wilcoxon test, respectively.

**Results:**

Irrespectively of the protocol adopted, 82.2% of the lesions were benign. 14.0% of the lesions were malignancies (5.1% of BI-RADS 3, 5.3% of BI-RADS 4A, 25% of BI-RADS 4B, and 83.3% of BI-RADS 4C lesions). 3.7% of the biopsies were precursor lesions. There was no evidence of underestimation in either protocols. In the standard protocol, the preoperative/postoperative diagnoses were identical. In the extended protocol, the postoperative diagnosis was less severe than the preoperative in 55.5% of cases (55.5% vs. 0%, p = 0.029), and preoperative ADH was totally removed. The phenomenon of discrepancy between diagnoses was associated with larger volume removed (8.20 ± 1.10 vs. 3.32 ± 3.50 cm^3^, p = 0.037) and higher removed percentage of the lesion (97.83 ± 4.86% vs. 74.34 ± 23.43%, p = 0.024)

**Conclusion:**

The extended protocol seems to totally excise precursor lesions, with minimal underestimation. This might possibly point to a modified management of ADH lesions.

## Background

The increased use of mammograms has led to the frequent detection of breast lesions, which in turn require further evaluation [1,2]. The most common mammographic abnormalities found on screening examinations are microcalcifications, solid lesions, and asymmetric densities [3,4]. About 90% of women with abnormal results do not have breast cancer [5,6]. The Breast Imaging Reporting and Data System (BI-RADS) was recommended by the American College of Radiology in order to provide a common language to indisputably describe the degree of suspicion regarding a mammographic lesion [7-9]. BI-RADS 3 lesions are considered as probably benign with a risk for malignancy less than 2% [7-9]. Suspicious lesions with a substantial probability but without the classic appearance of malignancy are classified as BI-RADS 4 [7,9,10]. Biopsy should be considered in these lesions. BI-RADS 5 lesions are highly suggestive of malignancy. It is recommended that appropriate action should be taken [7-10].

To establish a preoperative diagnosis, excisional biopsies, core needle biopsies [11-14] and vacuum-assisted breast biopsies (VABB) have been used [15,16]. Vacuum-assisted breast biopsy with stereotactic guidance has become an important part of the work-up of patients with suspicious breast lesions. The improved quality of vacuum-assisted core biopsy specimens, superior calcification retrieval, and accuracy, are well described in the literature [15-18]. Apart from the management of lesions with microcalcifications, VABB is an effective method for the evaluation of non-palpable mammographic solid lesions without microcalcifications [19].

Nonetheless, VABB has the disadvantage of histological underestimation, which renders the management of atypical ductal hyperplasia (ADH), and ductal carcinoma in situ (DCIS) somewhat difficult [20]. Up to 50% of the lesions diagnosed as DCIS by VABB show foci of invasion on pathological examination after surgical resection of these lesions [21-23]. The underestimation of ADH ranges in the literature from 0–35% [22].

This prompts an interesting question: does the removal of larger volumes of breast tissue, obtained contiguously from a single site in the breast at directional vacuum-assisted 11-gauge needle biopsy, result in decreased underestimation?

To answer this question, we have developed a modified VABB procedure using a considerably greater number of cores and offsets. The main objective of our study was:

(i) to compare the extended way of performing VABB and the recommended way according to the consensus meeting in Norderstedt.

(ii) to investigate the putative effects of greater volume (cm^3^) excised on the management of non-palpable solid lesions without microcalcifications.

## Patients and methods

We present the Greek experience, since our Breast Unit is the only center equipped with VABB and a Fischer's table. The material of this study consisted of 107 procedures performed from January 2004 to September 2006 in our Unit on women with a median age of 53 (range 37–77) years, (mean ± SD: 54.25 ± 9.58) for non-palpable solid lesions of the breast without microcalcifications.

Within this period, 355 women with non-palpable mammographic findings successfully underwent VABB; 107 biopsies were performed for mammographic solid tumor without microcalcifications (239 biopsies for microcalcifications and 9 for asymmetric density).

Before VABB, all patients were evaluated by one of the two radiologists of our Unit and a BI-RADS category was assigned. For lesions categorized as BI-RADS 3, follow-up was generally recommended. However, VABB was performed in the cases where family history was strongly positive or when the patient and referring physician expressed particular concern. In our unit, most of the BI-RADS category 5 cases are directly submitted for surgical biopsy in view of the great likelihood of cancer.

During this period of time, VABB was performed by 5 surgeons. In all instances, a radiologist was present to assist in the targeting. All women were informed about the procedure by the surgeon performing the intervention. The Mammotome biopsy was performed on a digital prone table (Mammotest, Fischer Imaging, Denver, CO, USA), using 11-gauge Mammotome vacuum probes, under local anesthesia.

Women who underwent VABB were randomly allocated to two standard protocols. The first category (recommended protocol) included 54 women. We attempted to obtain 24 cores from these women, according to the consensus meeting in Nordesterdt. For this purpose, we used 1 offset-main target in the middle of the lesion and one offset inside the solid lesion. 12 cores were excised from each offset (12*2 = 24).

In the second category (extended protocol), which included 53 women, we used one offset-main target in the middle of the solid lesion and 7 peripheral offsets, each one corresponding to a vertex of the hypothetically inscribed canonical heptagon in the round lesion, i.e. approximately 51 degrees away from each other. 12 cores were excised from each offset [(7+1)*12 = 96] in each patient of this group.

In both groups, a clip was placed in the biopsy site. After VABB, a mammogram on the women confirmed the excision of the suspicious non-palpable solid tumor, showing the cavitation in the suspicious area. The cores were examined by a pathologist and a preoperative diagnosis was established.

Afterwards, all women (except for one woman in the standard protocol group, to whom chemotherapy was administered, because NHL was diagnosed) with a preoperative pathological diagnosis of ADH, DCIS, LN, or invasive carcinoma, underwent open surgery under general anesthesia using a hook-wire. A second pathologist, blind to the preoperative results and to the protocol performed on the patient, examined the tissue removed by the surgeon. Thus, a second, postoperative diagnosis was independently and blindly made.

The percentage of the surface excised via VABB was retrospectively calculated on the mammogram, in the operated women of both protocols. The remaining surface was measured by calculating firstly the surface (cm^2^) of the solid tumour. More precisely, we measured the two dimensions of the solid tumour from the *face *mammogram using a ruler. Using the same method, we estimated the surface of the solid lesion that was left after VABB, using the formula for the area of circle segments. The subtraction of the above surfaces yielded the surface of the lesion excised. Also, the volume of specimens excised by VABB was determined in these patients.

The association between pathological diagnosis and BI-RADS classification, as well as the discrepancy between preoperative and postoperative diagnoses along with the protocol adopted were evaluated with the appropriate statistic, indicated in the results' section in parentheses. Statistical analysis was performed with STATA 8.0 statistical software.

Permission has been obtained from the local institutional review board for publication of the findings summarized in this study.

## Results

In 39 out of 107 women (36.4%) subjected to VABB, the non-palpable solid tumor was characterized as BI-RADS 3 (probably benign). In 68 out of 107 procedures (63.6%) the suspicious lesion was classified as BI-RADS 4. BI-RADS 4A subclassification is presented in Table 1.

**Table 1:** Non-palpable mammographic solid lesions: BI-RADS classification and preoperative diagnosis by VABB

	**Preoperative diagnosis**			
	**Benign**	**Precursor lesion**	**Malignancy**	***Total***

**BI-RADS 3 ***95% CI*	37 (94.9%)	0	2 (5.1%) *0.6%–17.3%*	39
**BI-RADS 4A ***95% CI*	34 (89.4%)	2 (5.3%)	2 (5.3%) *0.6%–17.8%*	38
**BI-RADS 4B ***95% CI*	16 (66.7%)	2 (8.3%)	6 (25%) *9.8%–46.7%*	24
**BI-RADS 4C ***95% CI*	1 (16.7%)	0	5 (83.3%) *35.9%–99.6%*	6

***Total***	88	4	15	107

The specimens obtained by VABB revealed a benign lesion in 88 out of 107 cases (82.2%), irrespective of their BI-RADS classification. The predominant diagnoses were fibrocystic changes, fibroadenoma, and adenosis/sclerosing adenosis; 32 out of 88 cases (36.4%) were fibrocystic changes, 18 lesions out of 88 (20.5%) were fibroadenomas, and 22 out of 88 lesions were adenoses/sclerosing adenoses (25%). Epitheliosis with atypia (5.7%), papillomas (4.5%) and haemangiomas (2.3%) were less common.

According to the pathological evaluation of the tissue samples obtained by VABB, in 15 out of 107 cases (14%), a malignancy was found, irrespective of the BI-RADS classification; 3.7% of the cases (4 out of 107) were precursor lesions [two cases of ADH and two cases of lobular neoplasia (LN)] (Table 1). Ductal invasive carcinoma was the predominant type (14 out of 15 malignant cases, 93.3%)

Irrespectively of the protocol adopted, there was a statistically significant increase in the probability of malignancy along with the increasing severity of the BI-RADS classification (p < 0.001, malignancy vs. all other diagnoses; Fisher's exact test).

The random allocation of cases to the two protocols is depicted in detail in Table 2. No statistically significant differences were documented in the frequency of cancer between the two protocols.

**Table 2:** Allocation of cases to the two protocols.

	**Preoperative diagnosis**						
	**Benign**		**Precursor lesions**		**Malignant cases**		***Total***

	*Standard protocol*	*Extended protocol*	*Standard protocol*	*Extended protocol*	*Standard protocol*	*Extended protocol*	

**BI-RADS 3**	20	17	0	0	2	0	39
**BI-RADS 4A**	16	18	1	1	1	1	38
**BI-RADS 4B**	8	8	1	1	3^a^	3	24
**BI-RADS 4C**	0	1	0	0	2	3	6

***Total***	44	44	2	2	8	7	107

^a ^NHL was allocated to the standard protocol, but was not operated

As described above, for each patient with a precursor or malignant lesion [except for the patient with non-Hodgkin lymphoma (NHL), to whom chemotherapy was administered], two pathological diagnosis existed, one preoperative and one postoperative (Table 3). The pathological diagnoses were identical in 50% of cases (Table 4). Interestingly enough, there was no evidence of underestimation of the lesion by VABB. On the contrary, in 5 cases (all of them belonging to the extended protocol), the surgical diagnosis on the remaining tissue was less severe than that initially made by VABB. The above 5 cases comprised 4 cases of ductal invasive carcinoma and one case of ADH.(Table 3, in bold).

**Table 3:** Malignant and precursor lesions in the two protocols

**Protocol**	**BI-RADS**	**Diagnosis by VABB**	**Postoperative diagnosis**
Standard	3	IDC	IDC
Standard	3	IDC + LN	IDC + LN
Standard	4A	IDC + DCIS + LN	IDC + DCIS + LN
Standard	4A	ADH	ADH
Standard	4B	LN	LN
Standard	4B	IDC	IDC + DCIS
Standard	4B	IDC + DCIS	IDC + DCIS
Standard	4C	IDC	IDC
Standard	4C	IDC	IDC
			
Extended	4A	LN	LN
Extended	4A	IDC	**Papilloma**
Extended	4B	ADH	**Normal mammary tissue**
Extended	4B	IDC	**ADH**
Extended	4B	IDC + DCIS	IDC + DCIS
Extended	4B	IDC + DCIS	IDC + DCIS
Extended	4C	IDC	**ADH**
Extended	4C	IDC	IDC
Extended	4C	IDC	**Fibrocystic changes, apocrine metaplasia, epitheliosis**

**Table 4:** Summarized results in the two protocols

	**Offsets**		
	2–3 offsets	8 offsets	***Total***

**Identical results from both procedures**	9	4	13
**Cases in which the blind pathological examination after surgery yielded a less severe diagnosis**	0	5	5

***Total***	9	9	18

The phenomenon of less severe postoperative diagnosis was more frequent in the extended protocol (p = 0.029; Fisher's exact test) (Table 4). As expected, less severe diagnosis based on the surgical specimen was associated with larger volume removed by the VABB (p = 0.037; Mann-Whitney-Wilcoxon test for independent samples) and the higher removed percentage of the lesion (p = 0.024; Mann-Whitney-Wilcoxon test for independent samples) (Table 5).

**Table 5:** The effect of volume – percentage removed by VABB

	**Cases yielding identical results from both procedures**	**Cases in which the blind pathological examination after surgery yielded a less severe diagnosis**
**Volume removed by VABB **(Mean ± SD)	3.32 ± 3.50 cm^3^	8.20 ± 1.10 cm^3^
**Percentage of the lesion removed by VABB **(Mean ± SD, %)	74.34 ± 23.43	97.83 ± 4.86

Clinically significant haematoma developed in 5 out of 107 patients, 2 of them belonging to the standard protocol, and 3 to the extended protocol. None of them required surgical intervention. The rate of clinically important haematoma formation did not differ between the two protocols. Similarly, there was no statistically significant difference in the mean age of women between the two protocols.

Finally, in 3 additional procedures (i.e. 3 out of 110), VABB could not be performed, as the solid tumor was too close to the examination plate; these 3 cases have not been included the study.

## Discussion

The use of VABB on the Fischer's table has been proven effective for preoperative diagnosis of breast cancer [15], with very satisfactory sensitivity, specificity, positive and negative prognostic value [19]. Many studies have focused on the role of VABB in the assessment of lesions with microcalcifications [18,24]. Despite controversies, the role of VABB in the management of lesions without microcalcifications [21,25] has been documented.

The main disadvantage of VABB is underestimation [20-22]. The underestimation rate ranges in ADH between 0–35%, and in DCIS raises up to 50% [22,23]. It is not clear in the international literature whether the underestimation rate is higher in solid lesions or in microcalcifications [20-22]. The suspicion for underestimation has prompted to surgical intervention in the cases diagnosed as ADH, LN by VABB [20,26]. Thus, the need for a procedure minimizing or even eliminating the underestimation problem seems to be crucial in the clinical practice.

Interestingly, underestimation was not detected in either protocol. In the standard protocol group, the relatively small sample may account for this observation. However, the excision of 96 cores implies a totally different context, i.e. that of a quasi-total excision of the non-palpable solid lesion. The quasi-total excision seems to encompass two constituents: lack of underestimation and frequently no residual precursor/preinvasive/invasive tissue being left after VABB (as documented by the postoperative examination). No residual tissue after VABB has been occasionally reported in the literature [27-29], but has not been extensively studied.

With respect to the first constituent, total elimination of underestimation might seem intriguing, for various reasons. Results with larger (8G) needles have not led to the diminishment of underestimation [30,31]. Indeed, the needle size may be a crucial factor, but the setting of offsets and the number of cores excised may also be an important factor determining underestimation. A hint to this direction dates back to 2001, when Jackman *et al*., showed that obtaining of more than 10 specimens per lesion leads to reduced underestimation of DCIS [32]. Although a contradictory study exists [33], the latter adopted an upper limit of 20 specimens per case. As a result, the context of 96 cores has remained unexplored, to which the previous results cannot be projected with certainty.

Independently, it has been suggested that the radiological disappearance of the lesion cannot enable the total elimination of underestimation, in the context of 18 cores (range: 5–64 cores) [22]. However, it should be kept in mind that radiological disappearance is not a synonym of pathological disappearance. The context of 96 cores might in fact go beyond the radiological disappearance, which nevertheless is not totally reliable in lesions without microcalcifications, as those examined in this study. At any case, the sample size of our study is relatively small, and more studies are undoubtedly needed to precise the exact, putative percentage of underestimation which might emerge in a large series, when 96 cores are excised.

With respect to the second constituent, i.e. the lack of residual tissue, Liberman *et al., *(1998) have stated that the complete removal of the mammographic lesion does not ensure the complete excision of carcinomas [28]. Indeed, the fact that free surgical borders cannot be achieved does not permit any therapeutic implications with respect to carcinomas. Of notice however, the whole cancerous lesion was excised via VABB in 56% of the extended protocol cases, while there was sufficient invasive tissue for the postoperative diagnosis of IDC in all cases assigned to the standard protocol.

On the other hand, a quasi-total excision of ADH might be a different case. When 96 cores were obtained, underestimation was not observed; this implies that a diagnosis of ADH seems accurate, not harboring an underlying carcinoma. Indeed, the percentage of ADH in our series (1.9%) was surprisingly low compared with the international literature on non-palpable solid tumors [17-20,34,35]. That was probably due to the lack of underestimation, i.e. no DCIS cases were mistakenly added to the ADH group.

In parallel, given the total excision in the case of ADH, interesting implications with respect to ADH management arise. It would be tempting to recommend follow-up for ADH cases after the performance of the extended protocol, as accurate diagnosis plus total excision seem a particularly attractive. At any case, more studies are needed to ascertain whether the current management of ADH might be modified. Liberman *et al., *(2002) have shown that to 'excise' of to 'sample' a lesion does not exert a significant effect on ADH underestimation; however, the distinction between 'excision' and 'sampling' was made within a range of less specimens, i.e. between 4 and 47 (median 15) specimens [36]. Nonetheless, and given that recent studies on MRI-guided VABB with 9G needle did not diminish underestimation of ADH [37], ADH still remains an entity on which extended protocols should be tested.

Commenting on the population on which this study was based, most of the Mammotome biopsies were performed on BI-RADS category 4 patients, the most common indicator for the procedure [38,39]. The percentage of BI-RADS 3 lesions was relatively high due to various reasons. Firstly, because we are the only referral center in Greece to which patients with a higher risk or positive family history are sent from other Centers. Secondly, in many cases this is due to the woman's persistence. The repeated examinations at a 6-month interval and the re-evaluation of the finding caused them such anxiety, depression, and decrease in their quality of life, that they insisted on a VABB.

In our sample, 86% of the women that underwent VABB did not have breast cancer. All these women could have potentially undergone unnecessary open surgery. The malignancy rates were in accordance with the BI-RADS classification, and exhibited a statistically significant increasing trend along with the BI-RADS subgroups. According to the literature, the frequency of cancer in BI-RADS 3 lesions ranges from 0.5% to 2% [7,8]; in our sample, the observed frequency of malignancies was 5.1%. Given the confidence intervals of the proportion (Table 1), this discrepancy is not statistically significant and should be attributed to the relatively small size of the sample.

Finally, an important issue which should be addressed is the complication rate. More cores obtained could be associated with a higher degree of complications. However, in this study, VABB was well tolerated by the patients, and there was no significantly higher percentage of haematoma in the extended protocol. Larger studies monitoring the safety of the extended protocol seem though indispensable.

## Conclusion

This study presents and standardizes a new way of performing VABB which seems capable of reducing underestimation. The use of one main and 7 peripheral offsets, the excision of 96 cores, and the greatest excised volume might also possibly point to a modified management of women with ADH lesions. Further, comparative studies on larger series are needed to evaluate and possibly establish the extended VABB protocol.

## Abbreviations

VABB = Vacuum-assisted breast biopsy

ADH = atypical ductal hyperplasia

DCIS = ductal carcinoma in situ

BI-RADS = Breast Imaging Reporting and Data System

LN = lobular neoplasia

NHL = non-Hodgkin lymphoma

IDC = invasive ductal carcinoma

## Competing interests

The author(s) declare that they have no competing interests.

## Authors' contributions

**GCZ**: conception of the idea and design of the study, supervisor of VABB procedure, responsible for the critical setting of offsets in all patients

**FZ**: writing of the manuscript, evaluation of the analyzed findings, critical interpretation of the study results with respect to the international literature

**TNS**: statistical analysis, writing of the manuscript

**DK**: evaluation of all mammograms, assignment of BI-RADS categories, assisting at the targeting of the lesion

**AN**: contributing to the design of the study, critical evaluation of pre-operative specimens, establishment of pre-operative diagnosis

**VO**: comparison of pre-operative and post-operative diagnosis, critical evaluation of all discrepancies

**PD**: VABB performance in both protocols

**MK**: VABB performance in both protocols

**CF**: revising the manuscript for important intellectual content

**JB**: revising the manuscript for important intellectual content, gave the final approval of the version to be published
